# Dual Role of TNF and LTα in Carcinogenesis as Implicated by Studies in Mice

**DOI:** 10.3390/cancers13081775

**Published:** 2021-04-08

**Authors:** Ekaterina O. Gubernatorova, Almina I. Polinova, Mikhail M. Petropavlovskiy, Olga A. Namakanova, Alexandra D. Medvedovskaya, Ruslan V. Zvartsev, Georgij B. Telegin, Marina S. Drutskaya, Sergei A. Nedospasov

**Affiliations:** 1 Engelhardt Institute of Molecular Biology, Russian Academy of Sciences, 119991 Moscow, Russia; almpolinova@eimb.ru (A.I.P.); petropavlovskiymm@eimb.ru (M.M.P.); namakanova@eimb.ru (O.A.N.); medvedovskaya@eimb.ru (A.D.M.); zvartsev@eimb.ru (R.V.Z.); 2 Department of Immunology, Faculty of Biology, Lomonosov Moscow State University, 119234 Moscow, Russia; 3 Center for Precision Genome Editing and Genetic Technologies for Biomedicine, Engelhardt Institute of Molecular Biology, Russian Academy of Sciences, 119991 Moscow, Russia; 4 Branch of Shemyakin-Ovchinnikov Institute of Bioorganic Chemistry of the Russian Academy of Sciences (BIBCh, RAS), 142290 Pushchino, Russia; telegin@bibch.ru; 5 Sirius University of Science and Technology, Federal Territory Sirius, 354340 Krasnodarsky Krai, Russia

**Keywords:** tumor necrosis factor, lymphotoxin alpha, TNFR2, LTβR, cancer, microbiota, mouse models

## Abstract

**Simple Summary:**

Tumor necrosis factor (TNF) and its closely related cytokine, lymphotoxin alpha (LTα), are part of the TNF superfamily and exert their functions via both overlapping and non-redundant signaling pathways. Reported pro- and antitumorigenic effects of TNF and lymphotoxin are often context-dependent and may be contingent on a particular experimental approach, such as transplantable and chemically induced tumor models; tissue and organ specificity; types of cells producing these cytokines or responding to them; and the genotype and genetic background of mice. Here, we review the mechanisms of TNF/LTα involvement in cancer promotion and suppression as studied in mouse models. We also discuss the impact of microbiota on tumor development and manipulations of the TNF/LT system, which may be effective as anti-cancer therapy.

**Abstract:**

Tumor necrosis factor (TNF) and lymphotoxin alpha (LTα) are two related cytokines from the TNF superfamily, yet they mediate their functions in soluble and membrane-bound forms via overlapping, as well as distinct, molecular pathways. Their genes are encoded within the major histocompatibility complex class III cluster in close proximity to each other. TNF is involved in host defense, maintenance of lymphoid tissues, regulation of cell death and survival, and antiviral and antibacterial responses. LTα, known for some time as TNFβ, has pleiotropic functions including control of lymphoid tissue development and homeostasis cross talk between lymphocytes and their environment, as well as lymphoid tissue neogenesis with formation of lymphoid follicles outside the lymph nodes. Along with their homeostatic functions, deregulation of these two cytokines may be associated with initiation and progression of chronic inflammation, autoimmunity, and tumorigenesis. In this review, we summarize the current state of knowledge concerning TNF/LTα functions in tumor promotion and suppression, with the focus on the recently uncovered significance of host–microbiota interplay in cancer development that may explain some earlier controversial results.

## 1. Introduction

**Figure 1:**
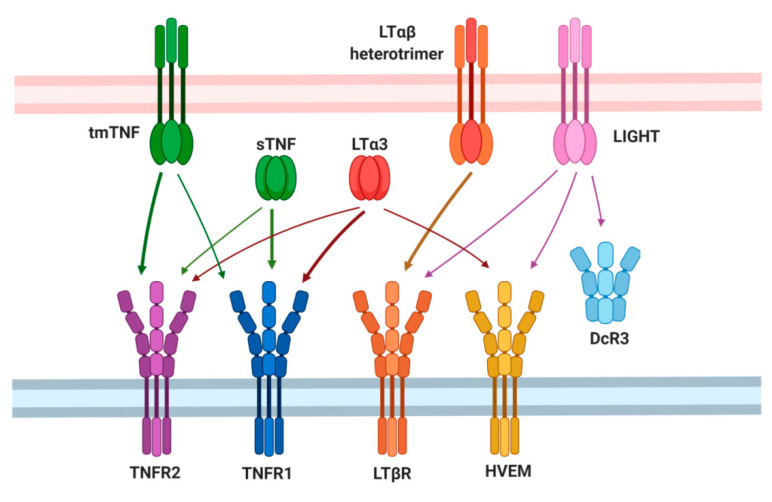
Ligands and receptors of the tumor necrosis factor (TNF)/lymphotoxin (LT) axis. Tumor necrosis factor (TNF) exists in either soluble (sTNF) or trans-membrane (tmTNF) form and inte-racts with its two receptors: TNFR1 (TNFp55) and TNFR2 (TNFp75). Another ligand for TNF receptors (TNFRs) is lymphotoxin α homotrimer (LTα3) that, in addition, binds herpes virus entry mediator (HVEM). HVEM, lymphotoxin β receptor (LTβR), and decoy receptor 3 (DcR3) are receptors for LIGHT, while LTβR also interacts with LTαβ heterotrimer, a membrane-bound form of lymphotoxin.

Tumor necrosis factor (TNF) and lymphotoxin alpha (LTα) exist as soluble homotrimers and interact with TNF receptors (p55 TNFR1 and p75 TNFR2). LTα homotrimer also binds to herpes virus entry mediator (HVEM) as does LIGHT (TNFSF14), another member of the TNF superfamily (Figure 1). In addition to their soluble forms, TNF homotrimer functions as a transmembrane molecule (tmTNF), whereas LTα homotrimer exists only in a soluble form, but may also act as a membrane-bound molecule by forming a heterotrimeric complex with LTβ (predominantly LTα1β2, but also LTα2β1) [1,2]. Transmembrane LTαβ heterotrimer and LIGHT signal via distinct LTβRs (lymphotoxin beta-receptors), whereas tmTNF predominantly interacts with p75 TNFR2. The primary role of TNF is immune regulation. TNF via its receptors activates multiple signaling cascades leading to induction of inflammation, cell death, or cell survival and is implicated both in cancer development and progression. LTαβ–LTβR signaling is the key pathway in the formation of lymph nodes and Peyer’s patches (PP), although it is not clear whether TNF may be absolutely required for the development of normal PP, since different TNF knockout strains either develop or lack PP [3,4,5,6]. Lymphotoxin has specific roles in the secondary lymphoid tissue’s organogenesis and in supporting lymphoid microenvironments, but also in host defense and inflammation. Thus, TNF and LTα are indispensable for maintaining immune system development and homeostasis; at the same time, these cytokines have distinct non-overlapping roles in inflammation.

The history of TNF is closely related to the history of cancer immunotherapy. William Coley was the first to use endotoxin-induced antitumor activity to treat inoperable sarcomas [7]. In the late 1960s, a cytotoxic factor, produced by lymphocytes in response to their interaction with specific antigens [8], or as a result of mitogenic stimulation, was described [9] and named lymphotoxin [10]. However, one cannot exclude that this cytotoxic substance contained not only LTα, but also TNF, the activity described in 1975 by Lloyd Old’s group as an endotoxin-induced serum factor that could cause tumor necrosis [11]. TNF and LTα genes were cloned a decade later [12,13,14,15] and when overexpressed showed similar cytotoxic and antitumor activity. This remarkable antitumor activity of TNF, however, turned out to be of limited use in patients due to its systemic toxicity [16]. On the contrary, systemic inhibition of TNF demonstrated striking therapeutic effects in the treatment of several autoimmune diseases [17]. At the same time, continuous use of systemic anti-TNF/anti-LTα biologics, such as Etanercept, could result in increased risk of cancer development [18]. This review summarizes findings regarding the role of TNF and LTα in transplantable and chemically induced mouse cancer models and discusses some unresolved controversies.

## 2. TNF/LT and Lung Cancer

Lung cancer is the most commonly diagnosed and deadly type of cancer. The influence of endogenous TNF on lung metastasis has mostly been investigated in mouse models using transplantable tumor cell lines (Table 1). In an experimental fibrosarcoma metastasis model, a single injection of low-dose recombinant human or mouse TNF prior to, but not after, fibrosarcoma inoculation increased the amount of lung metastasis, suggesting that TNF may enhance the vascular adhesion of tumor cells [19]. A similar effect was also observed in a B16F10 murine melanoma model of lung metastasis [20]. In another lung metastasis mouse model, renal adenocarcinoma (Renca) inoculation into TNFR1-deficient mice displayed spontaneous regression of metastasis foci as compared with wild-type (WT) mice, indicating that signal transduction via TNFR1 supports tumor neovascularization and promotes lung metastasis [21].

TNF-mediated signaling on tumor cells was also implicated in the process. Knocking down TNFR2 in Lewis lung carcinoma cells combined with a low dose of recombinant mouse TNF significantly inhibited carcinoma growth in WT mice, suggesting that TNF/TNFR2 signaling in tumor cells is pro-tumorigenic in this transplantable tumor model [22]. In line with this, TNFR2 expression by human non-small cell lung cancer tissue is related to a poor prognosis [38]. Chronic inflammation is an independent risk factor and a cancer hallmark [39]. In the case of lung cancer, a chronic obstructive pulmonary disease (COPD) is one of the most prominent risk factors. COPD patients have elevated levels of TNF in bronchoalveolar fluid (BALF) [40] and exhaled breath condensate [41], indicating a chronic inflammatory process in the lungs. TNF causes COPD and lung cancer promotion by supporting myeloid-derived suppressor cell (MDSC) accumulation within the tumor with subsequent tumor cell proliferation and increased angiogenesis. In particular, TNF overexpression in the airway epithelium of Kirsten rat sarcoma viral oncogene (K-ras) mutant mice, in the context of COPD-like inflammation, promoted lung tumor growth [42]. TNF is implicated in the secondary tumor resistance to epidermal growth factor receptor (EGFR)-blocking therapy by tyrosine-kinase inhibitors of non-small cell lung cancer. EGFR signaling actively suppresses TNF levels by decreasing TNF mRNA stability. Therefore, EGFR inhibition leads to an increase in TNF production that may trigger secondary lung cancer progression [43]. Taken together, TNF seems to have a tumor-promoting role in the case of lung cancer metastasis and inflammation-related tumorigenesis.

Regarding the role of LTα, a growing amount of evidence implicates lymphotoxin in antitumor activity. For instance, inoculation of B16F10 melanoma cells into LTα-deficient mice resulted in accelerated lung metastasis as compared with littermate control mice, presumably, due to impaired natural killer (NK) cell migration to the lungs [26]. Morever, in the same model, administration of a cancer-specific antigen antibody–LTα fusion protein enhanced eradication of pulmonary metastasis via an improved T-cell response evoked by LTα-dependent induction of peripheral lymphoid tissue at the tumor site [23]. This effect, most likely, was due to LTαβ–LTβR interaction, since LTβR blockade abrogated the formation of tertiary lymphoid structures in the lungs [44]. In addition, LTα production by effector T cells was shown to potentiate an antitumor response in a B16F10 melanoma pulmonary metastasis model [45], while blocking LTβR with a neutralizing monoclonal antibody decreased effector T-cell cytotoxicity in vitro [46], suggesting that LTαβ–LTβR interaction was crucial for the cytotoxic cell activation required for tumor regression. With respect to the role of LTβR in lung cancer development, published results remain controversial. Increased LTβR mRNA levels in tumor, but not in normal, tissues were associated with a worse overall survival in patients with lung adenocarcinomas [47]. LTβR expression in the lungs correlated with inflammation, at least in COPD [44]; therefore, increased LTβR expression in tumors may enhance inflammation and promote tumor growth. Moreover, stimulation of LTβR may subsequently trigger downstream non-canonical NF-κB signaling via activation of NF-κB-inducing kinase (NIK), which is involved in metastatic gene upregulation [48]. As already mentioned, LTβR is necessary for tertiary lymphoid structure formation in the respiratory tract [49]. Taken together, LTβR-mediated signaling may exert opposing effects on tumorigenesis, presumably due to its ability to initiate different pathways inside the cell depending on the context. On one hand, its increased expression may drive inflammation and upregulate metastatic gene expression; on the other hand, intact tertiary lymphoid tissue formation and cytotoxic cell accumulation are a part of the normal antitumor response.

## 3. TNF/LT and Skin Cancer

As already discussed, systemic TNF neutralization is a standard treatment for such autoimmune diseases as rheumatoid arthritis, psoriasis, and inflammatory bowel disease (IBD). However, one of the profound side effects of this therapy is the increased risk of skin cancer development, especially non-melanoma skin cancer [18,50,51]. Experimental data suggest that TNF can perform both pro- and antitumorigenic functions and the choice between the two alternatives is also context-dependent (Table 1, Table 2, and Table S1). On the one hand, published data suggest that TNF plays a deleterious role in a mouse model of chemically induced squamous cell carcinoma (Table 2).

**Figure 2:**
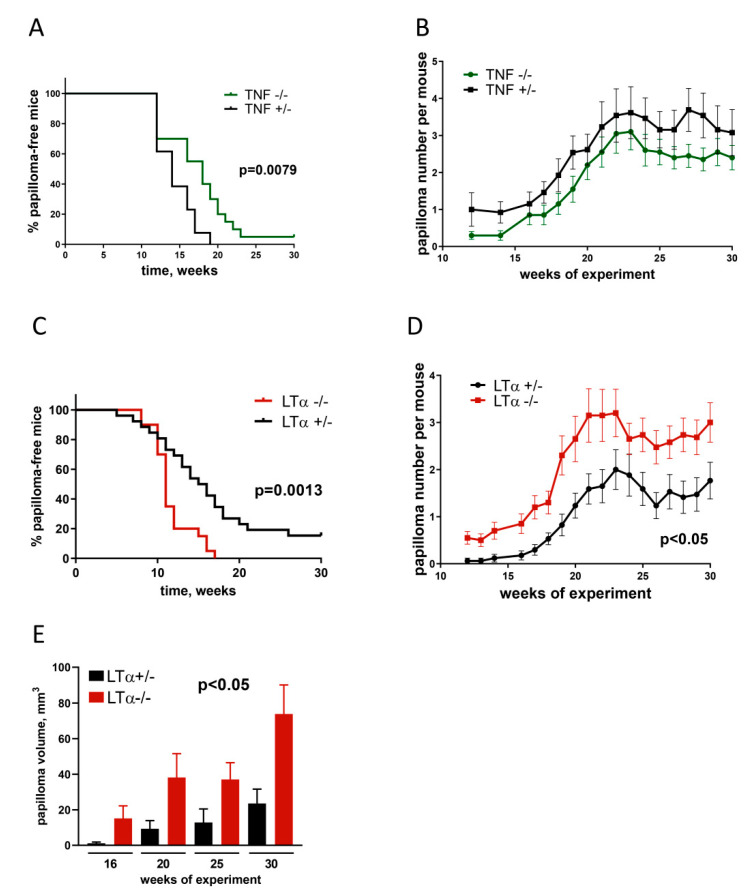
TNF-deficient mice with unperturbed LTα expression [3] are partially protected from DMBA/TPA skin carcinogenesis, while genetic LTα ablation sensitizes mice to this type of skin cancer. TNF-deficient mice were compared to heterozygous TNF^+/−^ co-housed littermate control mice. LTα-deficient mice were compared to heterozygous LTα^+/−^ co-housed littermate control mice. All animals aged 6–8 weeks were provided by the Unique Scientific Unit “Biomodel”, Branch of Shemyakin–Ovchinnikov Institute of Bioorganic Chemistry of the Russian Academy of Sciences (BIBCh, RAS), Pushchino, Moscow Region, Russia. The skin carcinogenesis was induced as previously described [66]. Briefly, a single application of 25 mcg of DMBA in 100 mcl of acetone was administrated on day 0 to the shaved back skin area. TPA was administered at 4 mcg per 100 mcl acetone to the same area three times a week for 20 weeks. After that, mice were further monitored for an additional 20 weeks and papilloma formation and size were measured on a weekly basis. (**A**). Percent of papilloma-free TNF-deficient and littermate control mice throughout the experiment. (**B**). Number of papillomas per TNF^+/−^ and TNF^−/−^ mouse throughout the experiment. (**C**). Percent of papilloma-free LTα-deficient and littermate control mice. (**D**). Number of papillomas per LTα^+/−^ and LTα^−/−^ mouse throughout the experiment. (**E**). Total papilloma volume per LTα^+/−^ and LTα^−/−^ mouse.

It should be noted that, in these earlier studies, co-housing and/or littermate control mice were not always used, making it difficult to exclude a possible impact of microbiota variation on the inflammatory response. On the other hand, more recent experiments in a TPA/DMBA two-step skin carcinogenesis model suggested that the difference in tumor load between TNF-deficient and co-housed littermate control mice may not be as dramatic as previously reported and is microbiota-dependent (Figure 2A,B). The need to use littermate control mice and/or cohoused mice, especially in cancer research, is supported by a number of studies [62,63]. External factors such as transport of mice, strain-specific alterations in host inflammatory responsiveness [64], or breeding-colony-dependent differences in commensal gut [65] and skin [66] microbiota may impact carcinogenesis. Administration of anti-TNF monoclonal antibodies enhanced the resistance of mice to chemically induced skin cancer [55]. In turn, genetic knockout of TNFR1 or TNFR2 was also associated with a reduced tumor number [58]. Additionally, selective elimination of TNF production by B-cells resulted in a decreased papilloma incidence, while B-cell transfer from DMBA/TPA-treated WT mice into TNF-deficient mice rescued tumor development, comparably to wild-type recipients [57]. This study, however, did not clearly indicate the use of littermate or co-housed mice in the control groups, suggesting that a difference in microbiota composition could be an additional tumor-promoting factor. Finally, B16F10 melanoma cells selected for low production of TNF demonstrated increased tumor growth and reduced necrosis in vivo in comparison with cells that did not produce TNF, whereas cells selected for a high TNF production did not have any advantage over control cells [25]. More evidence of antitumorigenic effects of TNF comes from a study involving athymic NCr-nu/nu nude mice inoculated subcutaneously with UV-induced skin cancer 1591-RE cells engineered to secrete hTNF and characterized by reduced tumor growth in comparison with non-transfected control cancer cells [27], suggesting an antitumor activity of TNF in vivo in the absence of T cells. Similarly, intraperitoneal or perilesional injections of recombinant mTNF or hTNF into mice inoculated with B16BL6 melanoma cells resulted in a delayed cancer development [24]. Of note, this antitumor activity of TNF was most likely mediated via TNFR1, since hTNF does not interact efficiently with murine TNFR2. Taken together, ample evidence implicates a dual role of TNF in tumorigenesis depending on the exact mouse model, the experimental context, tumor vs. immune cell origin of the cytokine, and the type of TNF receptor mediating the signal. The possible impact of microbiota will be discussed in the subsequent sections.

In turn, the role of lymphotoxin in the development of skin cancer has also remained somewhat controversial, primarily, due to the complexity of LT signaling (Figure 1). For instance, genetic knockout of LTα leads to enhanced tumor growth and elevates the risk of metastasis in C57BL/6 mice inoculated with B16F10 melanoma as compared with littermate control mice [26]. Furthermore, according to preclinical data, LTα coupled with chemotherapeutic drugs, especially platinum, can act as a cytotoxic agent in epithelial cancer cells [67]. At the same time, a phase IIb clinical trial failed to prove the efficacy of a therapeutic strategy based on administration of recombinant human LTα in combination with cisplatin and fluorouracil in patients with metastatic esophageal squamous cell carcinoma [68]. Moreover, recent findings show that LTα secreted by infiltrating lymphocytes may enhance glycolysis of epithelial cells in a PFKFB3-dependent manner through the classical NF-κB pathway and promote proliferation and migration of epithelial cells, which may contribute to aberrant angiogenesis in head and neck squamous cell carcinomas (HNSCCs) [69]. Additionally, LTαβ–LTβR signaling promotes activation of the alternative NIK–NF–κB2/RelB pathway, enhancing hepatocyte growth/scatter factor receptor MET-mediated cell migration in HNSCCs [48].

**Figure 3:**
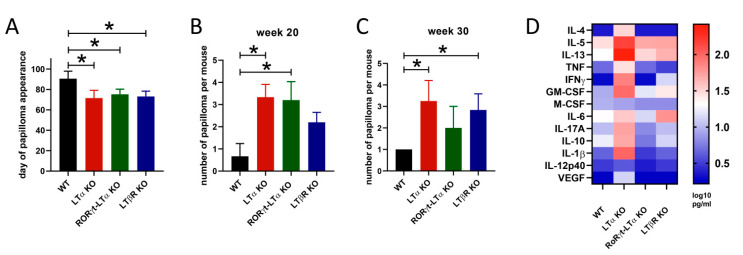
LTαβ–LTβR interaction protects mice from the development of DMBA/TPA chemically induced skin cancer. Mice [71,72,73] and the protocol for chemically induced skin carcinogenesis were previously described [66]. (**A**). Day of the first papilloma’s onset. * *p* < 0.05. (**B**). Number of papillomas per mouse at week 20 of the experiment. * *p* < 0.05. (**C**). Number of papillomas per mouse at week 30 of the experiment. * *p* < 0.05. (**D**). Heat map representing global variations in cytokine/chemokine responses (log_10_) in mice of different genotypes at week 20 of the experiment, as determined by multiplex analysis. Cytokine and chemokine levels in mouse sera were measured simultaneously using a multiplex microbead-based immunoassay, MILLIPLEX MAP Mouse Cytokine/Chemokine Magnetic Bead Panel-Premixed 32 Plex (MCYTMAG-70K-PX32, Merck) according to the manufacturer’s protocol.

Strikingly, in a DMBA/TPA-induced skin carcinogenesis mouse model LTα deficiency led to a dramatic increase in papilloma formation (Figure 2c–e), suggesting that LTα can actually protect mice from chemically induced skin cancer. To further study the role of lymphotoxin and to investigate the impact of LTαβ–LTβR signaling in cancer progression, skin tumors were induced in LTβR-deficient mice and in mice with LTα ablation restricted to RORγt^+^ cells. Both LTβR- and RORγt^+^ LTα-deficient mice developed more tumors as compared with co-housed littermate control mice (Figure 3), clearly implicating the key role of LTαβ–LTβR signaling in tumor control. Interestingly, the levels of serum cytokines, as revealed by multiplex analysis, indicated that LT-deficient mice developed the most prominent inflammatory response during the cancer induction phase (Figure 3D). However, since LTα-, LTβR-, and RORγt^+^ LTα-deficient mice all have abnormalities in the secondary lymphoid tissue formation [70,71], their susceptibility to chemically induced cancer may partially be associated with the delayed adaptive antitumor response.

**Figure 4:**
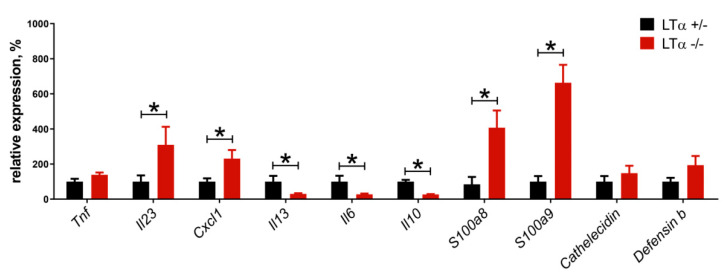
Relative expression of *Tnf, Il-23, Cxcl1, S100a8, S100a9, Il-13, Il-6, Cathelecidin,* and *Defensin b* in the LTα-deficient and control mouse skin two hours after the final phorbol myristate acetate (PMA) application. * *p* < 0.05. Acute skin inflammation was initiated in LTα-deficient and control mouse skin by administration of 4 mcg PMA in 100 mcl acetone on the shaved back skin area. Starting from day 2, the same amount of PMA treatment was followed by 25 mcg of Aldara cream application to the same area 10 min later with 4–6 cycles of PMA/Aldara every 2 days. Skin biopsies were collected, homogenized in TRK Lysis Buffer, and RNA extracted using an E.Z.N.A.^®^ Total RNA Kit (according to the manufacturer’s instructions). mRNA was transcribed into cDNA using a standard protocol and RevertAid First Strand cDNA Synthesis kit reagents followed by quantitative real-time PCR (see Table S2).

Chronic inflammation is strongly related to skin cancer progression. To evaluate the interplay between inflammatory cascades in the skin, proinflammatory cytokine expression, and cancer progression, a protocol for phorbol myristate acetate (PMA)-induced skin inflammation was used. The expression of IL-23, an IL-22-inducing cytokine [74], CXCL1, a neutrophil-attracting chemokine [75], and antimicrobial S100A8 and S100A9, which also may act as neutrophil-attracting molecules [76], was increased in the skin of LTα-deficient mice (Figure 4) following PMA application, indicating that LTα may indirectly participate in the control of neutrophil activation and recruitment. Finally, expression of IL-13, which has strong antitumor properties in the context of chemically induced skin cancer [66], was significantly decreased in the skin of LTα-deficient mice. Overall, these results suggest that the LTαβ–LTβR signaling axis is crucial for antitumor protection in the context of chemically induced skin cancer in mice. Further studies are required to establish the mechanism of this LTα-mediated protection.

## 4. TNF/LT and Liver Cancer

Hepatocellular carcinoma (HCC) often follows cirrhosis-driven inflammation of the liver [77] and may be triggered by hepatitis viruses [78,79]. TNF is a master regulator of liver inflammation as TNF-deficient mice are resistant to lipopolysaccharide/D-galactosamine-induced lethal liver toxicity [3,80]. The effect of TNF on hepatotoxicity is driven by TNFR1 and subsequent activation of NF-κB, but not by TNFR2 [81,82]. In the mouse model of spontaneous cholestatic hepatitis followed by HCC [83], the inflammatory process triggers hepatocyte NF-κB through upregulation of TNF. NF-κB inhibition through anti-TNF treatment resulted in apoptosis of transformed hepatocytes and failure to progress to HCC, indicating that TNF-induced NF-κB activation is essential for promoting inflammation-associated liver cancer [84]. The notion of the crucial role of NF-κB activation was further supported by a mouse study on the role of the lymphotoxin in liver cancer. To this end, LTαβ–LTβR interaction was shown to mediate angiogenesis by fibrosarcoma cells, thereby promoting tumor growth [28]. Moreover, the expression of LTαβ and LTβR was significantly increased upon Hepatitis B and C virus-mediated liver inflammatory response, and liver-specific overexpression of lymphotoxin induced liver inflammation and spontaneous HCC development, causally linking hepatic lymphotoxin overexpression to liver inflammation and liver cancer [85]. Taken together, these cytokines are pro-tumorigenic in the liver, and both TNF- and LT-mediated signaling contribute to liver inflammation, determining their ability to induce and promote liver cancer progression.

## 5. TNF/LT and Colorectal Cancer

Colorectal cancer (CRC) is the third most frequently diagnosed cancer and the fourth principal cause of cancer-related deaths worldwide [86]. There is a growing body of evidence demonstrating that TNF is crucial for CRC metastasis in the liver, the most common site for distant metastasis (Table 1) [87]. For instance, intrasplenic inoculation of BALB/c TNFR1-deficient mice with adenocarcinoma cells (CT26) resulted in a lower incidence of hepatic metastasis in comparison with WT counterparts [29]. Experiments with tissue-restricted knockout mice suggested that myeloid-cell-derived TNF plays an essential role in the suppression of colorectal liver metastasis. In particular, the metastatic load in the liver of mice subjected to liver ischemia after intrasplenic injection of CT26 cells correlated with the recruitment of monocytes with high TNF expression [30]. Furthermore, in this model TNF deficiency in Mlys^+^ myeloid cells was associated with enhanced hepatic tumor progression, including such antitumor effects of myeloid-cell-derived TNF as direct tumor cell apoptosis and a reduced expression of immunosuppressive molecules like TGF-β, IL-10, iNOS, IL-33, and heme oxygenase-1 [30]. More importantly, TNF may be involved in metastatic relapse following hepatic resection and consequent liver ischemia-reperfusion (IR) injury in patients with colorectal metastases. In particular, intraperitoneal injection of Etanercept in the model of IR induction resulted in a reduction in hepatic tumor number and size in BALB/c mice inoculated with CT26 cells [32]. However, pretreatment with murine recombinant TNF led to similar results, and was associated with decreased serum and hepatic TNF levels, and reduced liver injury after IR induction [32]. These contradictory results may be due to some preconditioning effect of TNF on liver cells; a low-dose TNF injection prior to hepatic injury promoted NF-κB activation, STAT3, cyclin D1, and cyclin-dependent kinase 4 expression, and cell cycle entry [88].

**Figure 5:**
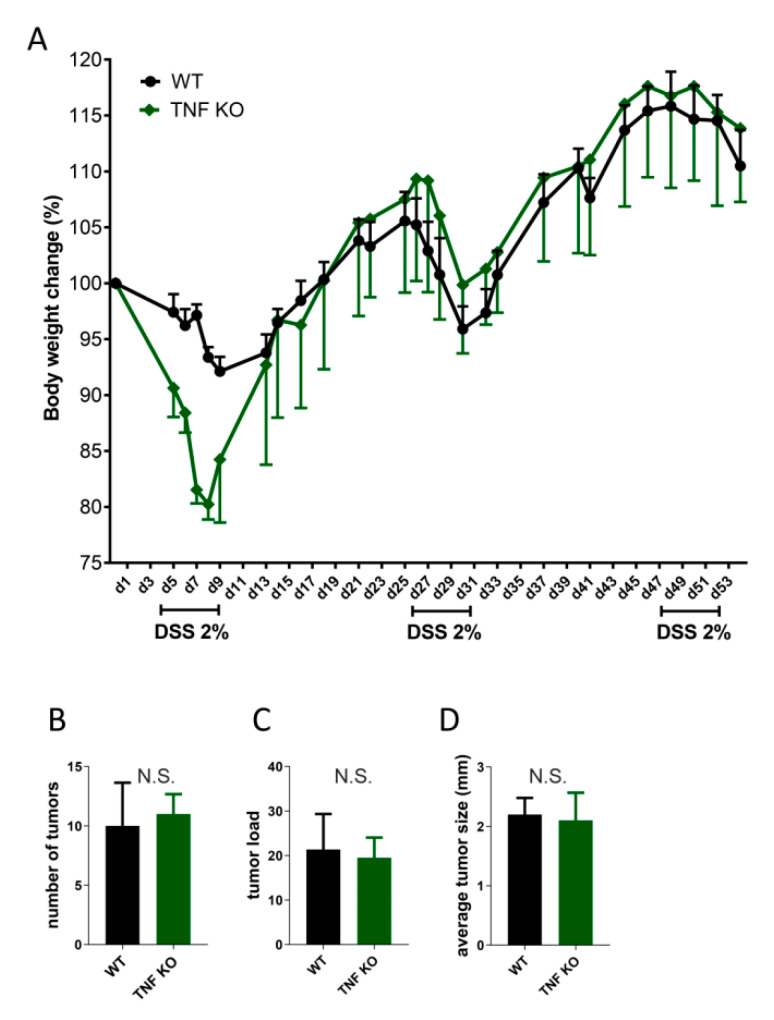
Genetic TNF ablation does not affect tumor incidence in the model of azoxymethane (AOM)/dextran sodium sulfate (DSS)-induced colorectal cancer following long-term co-housing with littermate control mice. TNF-deficient and heterozygous TNF^+/−^ littermate control mice received a single intraperitoneal (i.p.) injection of AOM (Sigma-Aldrich, Darmstadt, Germany) at a dose of 12 mg/kg body weight. A week after AOM injection, mice were given three cycles of 2% DSS in drinking water (Thermo Fisher) for five consecutive days followed by a two-week interval on drinking water without DSS. Body weight was measured throughout the experiment. After the last water cycle, mice were sacrificed and examined for polyp formation and colon length. (**A**). Percentage of initial body weight during the experiment. (**B**). Tumor number. *N.S.: not significant.* (**C**). Tumor load. *N.S.: not significant.* (**D**). Average tumor size. *N.S.: not significant.*

Previous studies demonstrated a pro-tumorigenic effect of TNF in a mouse model of chemically induced colorectal cancer (Table 2). In particular, BALB/c TNFR1-deficient mice treated with azoxymethane (AOM)/dextran sodium sulfate (DSS) developed fewer colon tumors with a smaller mean tumor size as compared with control mice [59]. Moreover, these results correlated with those obtained in the same experimental model of AOM/DSS colon cancer in wild-type BALB/c mice receiving intraperitoneal injections of Etanercept, which neutralizes both TNF and the soluble form of LT [59]. Similarly, weekly intraperitoneal injections of anti-TNF monoclonal antibodies starting at the end of the first DSS treatment were associated with a reduced tumor number and size in C57BL/6 mice [60]. Collectively, these data suggest that TNF may play an essential role in both CRC initiation and progression, at least in the AOM/DSS model, which, in turn, closely resembles the clinical course of human-colitis-associated CRC [89]. However, these results were not confirmed in recent experiments with co-housed littermate control mice. In particular, there was no difference between TNF-deficient and co-housed littermate control mice, neither in body weight change nor in tumor number and size, in the model of AOM/DSS-induced CRC despite increased inflammation in both groups (Figure 5). Such a discrepancy may be again explained by the long-term co-housing of experimental mice to minimize the effects of potential microbiota variation among mice of different genotypes, the phenomenon previously not addressed in earlier published studies (Table 1, Table 2, and Table S1) [90]. The notion that the role of TNF in tumor promotion may be microbiota-dependent is also supported by the fact that genetic knockout of TNF in p53-deficient mice does not alter their susceptibility to spontaneous lymphoma development in comparison with littermate control mice [91].

In turn, the role of lymphotoxin in the development of CRC is insufficiently studied, although the data available suggest that LTβR signaling can perform protective functions. In particular, intraperitoneal injections of anti-human LTβR agonistic monoclonal antibodies every 14 days resulted in reduced tumor growth in athymic nude mice subcutaneously inoculated with WiDr human colon adenocarcinoma, whereas a single injection of the anti-mouse LTβR agonistic monoclonal antibody caused tumor necrosis in BALB/c mice with CT26 colon carcinoma [33].

## 6. TNF/LT and Hematological Malignancies

Hematopoietic malignancies, such as leukemia, myeloma, and lymphoma, comprise eight to ten percent of all human malignancies. In the context of normal hematopoiesis, TNF was described as a T-lymphocyte differentiation factor [92], inhibitor of primitive hematopoietic progenitors [93], and hematopoiesis modulator in fresh umbilical cord blood [94]. TNF is a major regulator of demand-adapted hematopoiesis via signaling through TNFR1 [95]. Direct contribution of LT to hematopoiesis was not demonstrated; however, LT via LTβR controls the organization of the stromal microenvironment in hematopoietic niches. For instance, signaling via LTβR in bone marrow stromal cells by membrane LT is an important pathway for early NK cell development [96]. Finally, both TNF and LT may activate NF-κB, an important regulator of hematopoietic stem cell maintenance and homeostasis [97]. Nevertheless, several studies showed that, in addition to their homeostatic function, TNF and LT contribute to the onset and progression of hematopoietic cancers.

Leukemia derives from the bone marrow and is characterized by an overproduction of abnormal immune cells whose differentiation was arrested at a certain stage. The fitness of the abnormal cell pool depends on TNF, which regulates programmed cell death via TNFR1. TNF-deficient mice were partially protected from irradiation-induced apoptosis of bone marrow cells, indicating that TNF-mediated signal transduction is crucial for bone marrow cell death [98]. LIGHT–LTβR was recently shown to maintain the balance between self-renewal and differentiation of hematopoietic and leukemic stem cells [36]. In particular, LIGHT/LTβR signaling reduced cell cycling and protected hematopoietic stem cells from exhaustion. Similarly, LTβR deficiency reduced the number of leukemic stem cells and prolonged their survival in a murine model of chronic myeloid leukemia, supporting the idea that LTβR signaling in hematopoietic and leukemic stem cells mediates similar effects. Interestingly, leukemic-cell-derived TNF induced matrix metalloproteinase 9 (MMP-9) expression by the bone marrow microenvironment through TNFR1, thus contributing to acute lymphoblastic B-cell leukemia progression [34]. LTβR signaling may also modulate the leukemic microenvironment: inactivation of LTβR results in a significant delay in leukemia onset in TEL-JAK2 mice, which spontaneously develop T-cell leukemia, presumably due to the loss of LTβR signaling in thymic stromal cells [99]. Graft-versus-leukemia (GVL) constitutes an important part of anti-leukemic effects of transplantation and occurs when the donor marrow recognizes antigens on the leukemic blast cell as foreign and initiates immune-mediated clearance of malignant cells. It was shown that tumor clearance through GVL is dependent on both LTα and TNF expressed by donor cells, which induce apoptosis of recipient leukemic cells via TNFR1 [35].

Multiple myeloma (MM) originates in the bone marrow and affects plasma cells. Cancerous plasma cells produce faulty antibodies, which can damage kidneys and other organs and accumulate in the marrow. Progression of MM at early onset is driven by abnormally low levels of apoptosis, a high mitotic rate, and increased transendothelial migration of myeloma cells, partially mediated by TNF through NF-κB activation [100,101]. In line with this, myeloma-associated non-canonical genomic aberrations may reinforce pro-survival TNF-mediated NF-κB activity through autoregulatory RelB control and thereby exacerbate the disease [102]. Changes in the stromal compartment, such as angiogenesis [103] and stromal infiltrate, accompanying the development of multiple myeloma are also considered critical events in the progression of this disease [104,105]. For instance, bone marrow adipocytes, on one hand, produce factors that support myeloma cell growth and survival, and, on the other hand, produce adiponectin, which is myeloma-suppressive. It was shown that myeloma-cell-derived TNF downregulates adiponectin in bone marrow adipocytes, altering the bone microenvironment to support disease progression [106]. Finally, multiple myeloma progression reflects the escape of transformed plasma cells from T-cell recognition because of alterations in the expression of human leukocyte antigen (HLA) class I antigen processing–presenting machinery in transformed plasma cells [107].

Lymphoma usually starts in the lymphatic system and affects immune organs, causing lymphocyte accumulation in the spleen and lymph nodes. Lymphomas are divided into Hodgkin and non-Hodgkin lymphomas. Reed–Sternberg cells, a characteristic of Hodgkin lymphomas, are giant cells usually considered to be crippled germinal center B-cells. At the cell culture level, Reed–Sternberg-cell-derived LTα acts on endothelial cells to upregulate the expression of adhesion molecules that are important for T-cell recruitment into lesional lymph nodes in Hodgkin lymphoma [108], as well as for the maintenance of the inflammatory microenvironment [109]. The concept of cancer-cell-induced remodeling of the microenvironment as a mechanism of cancer progression was recently reinforced with the discovery of reciprocal interaction between *Myc-*driven lymphoma cells expressing vascular endothelial growth factor C (VEGFC) and LTα2β1 on one hand and the corresponding VEGF receptor-3 and LTβR on high endothelial venules on the other. These interactions caused vascular reprogramming, endothelial cell proliferation, and increased angiogenesis, which, in turn, resulted in reshaping of the lymphoma’s metabolism and acceleration of malignancy [37].

The pathogenic role of TNF was recently documented for clonal hematopoiesis of indeterminate potential (CHIP), an age-related condition characterized by a pool of early blood cell progenitors with a genetically distinct subpopulation of cells bearing unique mutations in DNA. This condition is usually caused by the emergence of an inactivating mutation in Tet methylcytosine dioxygenase 2 (Tet2) and is characterized by skewed myelomonocytic differentiation. It was found that chronic TNF exposure, as an “inflammaging” marker, favors the fitness of Tet2 mutant clones via conferring TNF resistance to sensitive bone marrow mutant progenitors [110]. TNF overexpression in the bone marrow niche may also suppress normal hematopoietic stem cells [111]. Thus, an increased TNF level, on one hand, establishes an inflammatory microenvironment that compromises normal hematopoietic stem cell renewal, and, on the other hand, provides an advantage to pathological clones. Taken together, the elimination of the chronic inflammation characteristic of inflammaging may represent a valuable therapeutic strategy for some hematological disorders.

## 7. TNFR2 in Cancer Progression

Accumulated data suggest that signaling through TNFR2 is important for tumor expansion since TNFR2 is indispensable to Treg cell functions [112] and the generation and survival of MDSCs [113]. Tumor cells may use immunosuppressive properties of Tregs and MDSCs to escape cytotoxic cell-induced cell death. Tumor infiltrates enriched for highly suppressive TNFR2^+^ Tregs and for increased numbers of TNFR2^+^ Tregs in the peripheral blood are predictors of a poor outcome in several types of cancer [114,115,116,117]. However, aberrant TNFR2 expression was also reported for tumor cells; for example, in esophageal squamous cell carcinoma [118], colorectal cancer [119,120], ovarian cancer [121], Hodgkin’s and non-Hodgkin’s lymphomas [122,123], and renal cell carcinoma [124,125]. Since TNFR2 is linked to cell proliferation and survival [126], overexpression of TNFR2 on tumor cells may exploit this growth receptor to enhance their proliferation [114]. For instance, TNF‒TNFR2 interaction on breast cancer cells promotes highly suppressive phenotypes of regulatory T-cells and, thereby, enhances tumor escape from immune surveillance [127]. In the case of Sezary syndrome, a rare form of cutaneous T-cell lymphoma, administration of human TNFR2 antagonistic antibodies led to the rapid death of TNFR2^+^ tumor cells and TNFR2^+^ Tregs with an increase in the T-effector cell fraction in experiments in vitro and ex vivo [128]. Finally, antagonistic TNFR2 antibodies could reduce soluble TNFR2 secretion more potently than in Tregs from healthy donors, suggesting that these antibodies may preferentially target the tumor microenvironment [129]. Therefore, antagonistic TNFR2 antibodies can modulate the tumor microenvironment, increase the number of T-effector cells, and eliminate highly suppressive TNFR2^+^ Tregs. Taken together, TNFR2 targeting alone, or in combination with other treatments, presents a promising strategy for cancer therapy, as it potentially may have fewer side effects and act more selectively than checkpoint inhibitors [130,131,132,133].

## 8. Peculiarities of the LT System and Cancer 

Determining the specific contribution of lymphotoxin to carcinogenesis is complicated by the fact that LT/LTβR signaling is indispensable to lymph node formation during embryogenesis [26,71]. In line with this, lymphotoxin- and LTβR-deficient mice do not develop lymph nodes and show other structural abnormalities in the secondary lymphoid tissue’s organization [26,70,71,134,135,136]. Because of the complexity of LT-mediated signaling (Figure 1), experiments with lymphotoxin-deficient mice are somewhat difficult to interpret unless they are supplemented with data in receptor-deficient mice or bone marrow chimeras. A number of studies support the involvement of lymphotoxin in carcinogenesis; however, its role remains controversial and depends on the specific cancer type [85,137]. Ablation of LTα in p53-deficient mice that spontaneously develop tumors did not affect the incidence of sarcomas and lymphomas, questioning the direct link between these types of cancer and lymphotoxin [91]. On one hand, recent results from the experiments with littermate wild-type control mice in microbiota-controlled settings suggest that LTαβ–LTβR interaction may protect mice from chemically induced skin cancer (Figure 2C–E and Figure 3), presumably by suppressing hyperinflammation that drives the development of skin cancer. On the other hand, in a transgenic adenocarcinoma mouse model spontaneously progressing to prostate cancer, LTα deficiency rescued tumor-reactive T cells and effectively reduced cancer incidence. Moreover, a short-term treatment of mice, predisposed to prostate cancer, with the fusion protein consisting of the extracellular domain of LTβR and Fc reduced the size of the primary tumors and completely prevented metastasis later in life through the expansion of T cells specific to tumor antigens [138], suggesting the pathogenic role of lymphotoxin-mediated LTβR signaling in cancer. Finally, activation of LTβR on fibrosarcoma cells by LTαβ-bearing lymphocytes was required for induction of angiogenesis and solid tumor growth [28]. The ability of LTβR to initiate both cytotoxic and cell-protective pathways makes it functionally similar to TNFR2. Both LTβR and TNFR2 lack classical death domains and depend on a TRAF2-binding domain that is responsible for NF-κB activation [139]. In turn, the non-canonical NF-κB signaling pathway may represent a limiting step in malignant cell transformation, as shown in patients with multiple myeloma [140]. Given the important role of lymphotoxin in the formation of lymphoid organs and control of cell death and proliferation, more sophisticated mouse models are required to dissect specific lymphotoxin functions in cancer.

## 9. Immune System–Microbiota Interactions in Cancer Progression—A Clue to Resolving Earlier Controversies?

The interaction between the microbiota and the host may not only promote immune system development and support its homeostasis [141], but may also affect cancer-promoting chronic inflammation. Indeed, the role of microbiota in cancer development and progression has been recently recognized [61,90]. Table 1 and Table S1 summarize previously published work with a focus on a potential role of microbiota [26,52,61,91,138,142]. Apparently, in most of the earlier studies littermate control mice or co-housing of mice were not fully appreciated. Strong evidence for the microbiota’s involvement in cancer modulation was found in gastrointestinal cancers that clearly demonstrated a significant impact of *Helicobacter pylori* on gastric cancer [143]. Chronic inflammation promoted by *H. pylori* led to a disturbance in Wnt/β catenin signaling in epithelium, contributing to increased tumor transformation [144]. In the mouse model of AOM/DSS-induced colorectal cancer, MyD88-deficiency resulted in increased tumor growth [145], suggesting that signaling downstream of Toll-like receptors may provide protection from colon tumors [146]. However, in spite of MyD88 involvement, TLR4^−/−^ mice are protected from the development of colorectal cancer [147], while TLR2^−/−^ mice appeared to be more susceptible to tumorigenesis [148], presumably due to the different microbiota composition. TNF is the central cytokine produced in response to TLR activation; thus, it is plausible that microbiota-induced TNF may regulate host–microbiota interactions in the gut. In line with this, a number of studies suggest that TNF can modulate the gut microbiota’s composition, as TNF is increased in the mucosa, serum, and stool of patients with Crohn’s disease [149] and IBD [150,151]. Furthermore, anti-TNF therapy may be beneficial in gastrointestinal disorders [152], in part by modulating the microbiota’s composition. The interplay between TNF and microbiota was studied in animal models of colitis. TNF^−/−^ mice with trinitrobenzene sulfonic acid (TNBS)-induced colitis demonstrated less severe inflammation than WT mice [153]. Deep sequencing of 16S rRNA genes of fecal microbiota from TNF^−/−^ and WT mice before and after TNBS and sham treatment revealed that WT mice have a higher *Firmicutes* to *Bacteroidetes* ratio than TNF^−/−^ mice [153]. Moreover, the proportion of *Turicibacter* in WT mice was increased as compared with TNF^−/−^ mice both prior to and after colitis induction, suggesting that TNF expression may affect the bacterial composition in the gut. Finally, a recent study showed that attenuation of colitis-associated cancer due to anti-TNF treatment is microbiota-dependent, and that co-housed mice treated with anti-TNF together with untreated control mice may cancel the protective effect of anti-TNF therapy [61].

Published data support the idea that TNF modulates microbial composition and abundance and that microbiota can also affect TNF production. In the AOM/DSS model of colorectal cancer, probiotic introduction reduced the tumor burden via modulation of the immune response, including reduced TNF levels [154]. *Saccharomyces boulardii* was shown to reduce the TNF production in the AOM/DSS model and decrease the number of tumors [155]. In a randomized double-blind placebo-controlled trial of probiotics in the post-surgical period of colorectal cancer, a reduction in intestinal inflammation and production of pro-inflammatory cytokines, including TNF, were reported [156]. Microbial-specific indole 3-propionic acid (IPA) binding to its receptor on enterocytes resulted in downregulation of enterocyte-specific TNF, while the tight-junction proteins were upregulated. In the absence of the IPA receptor, mice were characterized by a leaky intestinal barrier and impaired microbiota control. Thus, microbial metabolites may directly modulate TNF production in enterocytes and support the barrier integrity indispensable to inflammation control [157]. Taken together, host–microbiota interactions require adequate control by the immune system, which is dependent on TNF production. Modulation of TNF levels by microbiota and vice versa may be an important component in the therapy of intestinal inflammation and inflammation-induced cancer.

LTα-mediated signaling plays an extremely important role in the control of microbiota; nevertheless, the functions of soluble LTα homotrimer are difficult to distinguish from those of the membrane-bound lymphotoxin LTαβ heterotrimer. Most of the reported lymphotoxin functions are related to its membrane-bound form [158,159]. However, recently, non-redundant functions of soluble and membrane-bound lymphotoxin in the production of IgA that affect intestinal microbiota composition were uncovered. Soluble LTα3 produced by RORγt^+^ ILCs (innate lymphoid cells) is necessary for T-cell-dependent production of IgA by B-cells in the lamina propria, regulating homing of T-lymphocytes into the intestine, while membrane-bound LTαβ on RORγt^+^ ILC is involved in the induction of T-cell-independent IgA production [73]. However, the impact of LTα–microbiota interaction on cancer progression remains largely unknown and should be addressed in the future.

## 10. TNF and LT as Prognostic Markers and Their Polymorphisms

TNF plays an important role in human cancer development and progression [160] and may serve as a prognostic factor. A large body of evidence highlights the association between TNF levels and the disease stage. In particular, TNF is increased in serum and in exhaled breath condensate of lung cancer patients [161], as well as in serum of patients with colorectal cancer [162] and in different types of leukemia [163,164]. In addition, salivary TNF is a potential prognostic biomarker for oral squamous cell carcinoma (SCC) [165]; moreover, TNF-mediated upregulation of SOD-2 supports cell proliferation and cisplatin resistance in esophageal SCC patients [166]. Similarly, TNF is overexpressed in liver tumor biopsies and predicts poor survival of HCCpatients [167], which can be linked to TNF-dependent c-myc expression via the induction of pituitary tumor transforming gene 1 [168] and to a positive feedback loop between TNF/p38 MAPK (mitogen-activated protein kinase) signaling and oncogene cathepsin C [169].

A growing body of data suggests that TNF is involved in the regulation of a tumor’s metastasizing ability. For instance, TNF is expressed at the invasive front of colon cancers [170]. Moreover, the effect of TNF on cancer invasiveness is tightly linked to the regulation of the miRNA expression. In particular, TNF-induced expression of miR-146a leads to the Merlin protein’s inhibition and to enhanced metastasis in human lung adenocarcinoma [171]. In addition, TNF is involved in colorectal cancer progression via the induction of miR-105, which modulates the epithelial–mesenchymal transition [172].

In turn, the LTα expression was found to be significantly higher in HNSCCs as compared with adjacent normal tissues [69] and may promote tumor angiogenesis by reshaping the metabolism and enhancing glycolysis in endothelial cells in a PFKFB3-dependent manner [69]. Similarly, LTβ is overexpressed in chronic lymphocytic leukemia [173] and in cancers arising within lymphoid oropharyngeal and tonsillar sites [48]. At the same time, LTβR is amplified or overexpressed in HNSCCs of the larynx or oral cavity [48] and increased LTβR expression is associated with a worse overall survival in patients with non-small-cell lung cancer [47]. Nevertheless, LTβR signaling can suppress colorectal cancer development via the induction of IL-22bp, the level of which is downregulated in tumor tissues from patients with CRC and correlates with a poor prognosis [31].

Multiple trials of cancer treatment based on TNF and its derivatives (L19TNF, CNGRC peptide-TNF conjugate (NGR-TNF)) were carried out for melanoma and other solid tumors (Table S3). Other studies, on the contrary, investigated TNF blockers as potential drugs for complications (for example, in pneumonia following bone marrow transplantation) (Table S3). Despite much evidence correlating TNF with cancer severity and prognosis, clinical trial outcomes are not conclusive. For example, some trials of isolated limb perfusion with TNF in melanoma patients did not demonstrate a significant improvement in the TNF-treated group [174], whereas others reported some positive effects [175]. However, treatment of certain autoimmune disorders with TNF antagonists suggested that TNF can take part in tumor suppression. In particular, anti-TNF therapy increased the risk of lymphoma development in IBD patients [176,177,178].

The results of meta-analyses revealed several polymorphisms of TNF and LTα genes as predictive markers for malignancy progression. The TNF rs1800629 and rs1799724 polymorphisms may be linked to increased HCC development in non-Asian populations [179,180]. Similarly, the LT-α G/G variant rs909253 is of higher risk for HCC progression in the Chinese Han population in southern Taiwan [181]. Other studies showed that the rs1800629 G > A polymorphism in the TNF gene is associated with an increased risk of lung cancer, especially among Asians [182] and Tunisians [183]. Notably, the LTα C804A polymorphism was found to correlate with lung cancer in males [184]. As previously reported, the TNF rs1800629 G > A and G variants were indicated to affect skin basal cell carcinoma in Polish and Caucasian populations, respectively [185,186]. Given the importance of TNF in colorectal cancer progression, the polymorphisms of TNF rs361525 G > A and rs1800629 G > A were shown to correlate with the risk of CRC in Caucasians and Asians [187]. Studies that addressed the relationship between TNF polymorphisms and hematopoietic malignancies suggested that the TNF rs1799724 C > T polymorphism may be a causative factor for multiple myeloma [188], while TNF rs1800629 is a risk factor for diffuse large B-cell lymphoma in the Caucasian population [189] and non-Hodgkin lymphoma among Caucasians and Africans [189,190]. In turn, the LTα rs909253 A > G and AA genotypes were negative prognostic factors in leukemia [191]. In conclusion, because of the uneven distribution of different allele variants in populations, more studies involving epidemiological and etiological cases are needed.

## 11. Conclusions

TNF and LT were discovered as cytotoxic substances and are considered to be promising agents for cancer therapy. With our understanding of the complexity of TNF and LT signaling, this initial concept has undergone significant changes. We now know that the pro- and antitumorigenic effects of both TNF and LT depend on the specific conditions, on the type of producer and responder cell, and on the microenvironment. A better understanding of these mechanisms in the existing mouse models in microbiota-controlled settings may provide us with further insights into the role of these cytokines in cancer development, but also define the conditions and targets for successful anticancer therapy.

## Figures and Tables

**Table 1:** Anti- and pro-tumorigenic effects of TNF and LT, as implicated by transplantable mouse tumor models.

Transplantable Tumor Cell Type and Injection Site		Genetic Background of Recipient Mice	Additional Experimental Procedures	Reported Phenotype	Ref.
Meth A sarcoma	s.c.	(BALB/c x C57BL/6) F1 hybrid	Single administration of TNF-positive serum (i.v.)	Hemorrhagic tumor necrosis	[11]
CFS1-fibrosarcoma	i.v.	C3H/He, DBA/2	Single injection with rhTNF or mTNF (i.p.) 5 h before or 1 h after tumor cell inoculation	Enhanced lung metastasis, dose- and time-dependent effect	[19]
Renca RCC	i.v.	TNFR1 knockout in BALB/c	None	Regression of lung metastasis	[21]
B16F10 melanoma	i.v.	C57BL/6	Single injection with rmTNF (i.v.) 1 h before tumor cell inoculation	Enhanced lung metastasis	[20]
TNFR2^–/–^LLC	s.c.			No effect	[22]
			Low-dose injections with rmTNF (i.t.) for 6 days	Tumor regression	
LLC	s.c.		Low-dose injections with rmTNF (i.t.) for 6 days	Increased tumor growth	
GD2-expressing B16 melanoma	i.v.		Daily injections with αGD2–LTα fusion protein (i.p.) for 5 consecutive days	Reduced growth and number of lung metastasis foci	[23]
	s.c.		Daily injections with αGD2–LTα fusion protein (i.v.) for 7 consecutive days	Tumor flattening and necrosis	
B16BL6 melanoma	s.c.		Daily injections (i.p. or p.l.) with mTNF or rhTNF	Reduced tumor growth	[24]
Low TNF-expressing B16F10 melanoma or LLC	s.c		None	Enhanced tumor growth, reduced necrosis	[25]
High TNF-expressing B16F10 melanoma or LLC	s.c.		None	No effect	
TNF-expressing B16F10 melanoma or LLC cells	s.c.	TNFR1/TNFR2 double knockout in C57BL/6	None	No effect in the case of B16F10 and even reduced tumor growth in the case of LLC (compared with control cells)	
B16F10 melanoma	s.c.	LTα knockout in C57BL/6		Enhanced tumor growth	[26] *
B16F10 melanoma or LLC	i.v.		None	Increased incidence of metastasis	
hTNF-expressing murine 1591-RE cells	s.c.	athymic NCR nude mice	None	Reduced tumor growth	[27]
BFS-1 fibrosarcoma	i.d.	LTα/LTβ double knockout in C57BL/6	None	Reduced tumor growth in both cases	[28]
sLTβR-Fc fusion protein-expressing BFS-1 fibrosarcoma	I.d.	C57BL/6	None		
CT26 colorectal carcinoma	i.s.	TNFR1 knockout in BALB/c	None	Reduced incidence of liver metastasis	[29]
MC-38 colorectal carcinoma	i.s.	TNF^flox/flox^ LysM^cre/wt^ in C57BL/6	Hepatic ischemia-reperfusion injury	Increased liver metastasis	[30]
	i.c.	C57BL/6	Multiple injections with neutralizing LTβR-Fc fusion protein (i.p.)	Increased tumor number and load	[31]
CT26 colorectal carcinoma	i.s.	BALB/c	Single injection with etanercept or TNF (i.p.) followed by hepatic ischemia-reperfusion injury	Reduced liver metastasis	[32]
CT26 colorectal carcinoma	s.c.		Single injection with α-mLTβR agonistic Ab (i.p.)	Tumor necrosis	[33]
*Tnf* shRNA-expressing B-ALL	i.v.	C57BL/6	None	Increased survival	[34]
BCR/ABL myeloma	i.v.	C57BL/6 as donors, B6C3F1 as recipients	Transfer of BM from TNF, LTα or TNF/LTα double knockout mice into lethally irradiated mice	Increased survival, especially in the case of TNF/LTα double knockout mice	[35]
LTβR knockout BCR/ABL myeloma	i.v.	C57BL/6	None	Increased survival	[36]
Eμ-myc B-cell lymphoma	i.v.	C57BL/6	Two injections of α-mLTβR neutralizing Ab (i.p.)	Decreased tumor growth	[37]
		LtβR^flox/flox^ Cdh5^cre/ERT2^ in C57BL/6	None		

RCC—Renal cell carcinoma, LLC—Lewis lung carcinoma, s.c.—subcutaneous, i.v.—intravenous, i.t.—intratumoral, p.l.—paralesional, i.d.—intradermal, i.s.—intrasplenic, i.p.—intraperitoneal, rhTNF—recombinant human TNF, mTNF—mouse TNF, rmTNF—recombinant mouse TNF, hTNF—human TNF, TNF^flox/flox^ LysM^cre/wt^—tissue-specific genetic knockout of TNF in macrophages and monocytes, i.c.—intracecal, shRNA—short hairpin RNA, B-ALL—B-cell acute lymphoblastic leukemia, α-—anti-, LtβR^flox/flox^ Cdh5^cre/ERT2^—inducible tissue-specific genetic knockout of LtβR in endothelial cells. * littermate controls were used in this study.

**Table 2:** Anti- and pro-tumorigenic effects of TNF and LT, as implicated by chemically induced mouse tumor models.

Chemically Induced Cancer Mouse Model	Genetic Background	Additional Experimental Procedures	Resulting Phenotype	Ref.
DMBA/TPA-induced skin carcinogenesis	TNF knockout in 129/Svj (CD-1 mice as controls)	None	Reduced tumor number	[52] *
	TNF knockout in mixed 129Sv × C57BL/6 background or BALB/c	None		[53]
	TNF knockout in BALB/c	None		[54]
	TNF knockout in C57BL/6, 129/SvEv, BALB/c	None		[55]
	C57BL/6	Injections of α-TNF (i.p.) 1 day prior to DMBA treatment and once a week during TPA promotion		
	TNF knockout in C57BL/6	None		[56]
	TNF knockout in C57BL/6	None		[57]
	Tissue-specific B-cell TNF knockout in C57BL/6	None	Reduced tumor number, less pronounced effect	
	C57BL/6	Adoptive transfer of splenic B-cells from DMBA/TPA-treated WT mice into DMBA/TPA-treated TNF knockout mice	Increased tumor number compared with TNF knockout mice	
	TNFR1 or TNFR2 knockout in C57BL/6	None	Reduced tumor number, especially in TNFR1 knockout mice	[58]
DMBA/okadaic-acid-induced skin carcinogenesis	TNF knockout in 129/Svj (CD-1 mice as controls)	None	Reduced tumor number	[52] *
AOM/DSS-induced colorectal cancer	TNFR1 knockout in BALB/c	Daily injections of Etanercept (i.p.) from day 56 to day 60	Reduced tumor number and growth	[59]
	C57BL/6	Weekly injections of α-TNF (i.p.) following the first DSS cycle		[60]
	C57BL/6	Multiple injections of neutralizing LTβR-Fc fusion protein (i.p.)	Increased tumor number and load	[31]
Colibactin/DSS-induced colorectal cancer	APCmin/-in 129/SvE	Injections of α-TNF (i.p.) every other day for up to 6 times immediately after DSS	Reduced tumor number, no effect when co-housed with control mice	[61] *
		Cecal microbiota transplantation from colibactin/DSS-exposed mice treated with α-TNF to germ-free mice followed by DSS exposure	Reduced tumor number as compared to germ-free mice transplanted with cecal microbiota from colibactin/DSS-exposed mice treated with PBS	
Colibactin-induced colorectal cancer	APCmin/-IL-10 knockout in 129/SvE	Twice-weekly injections of α-TNF (i.p.) twice a week starting at 8 weeks after *Escherichia coli* gavage and until the endpoint	Reduced tumor number, no effect when co-housed with control mice	

α-—anti-, DMBA—7,12-dimethylbenz[a]anthracene, TPA—12-O-tetradecanoylphorbol-13-acetate, AOM—azoxymethane, DSS—dextran sodium sulfate, i.p.—intraperitoneal. TNF-deficient mice treated with 7,12-dimethylbenzanthracene (DMBA)/12-O-tetradecanoylphorbol-13-acetate (TPA) developed fewer skin papillomas as compared with control mice [52,53,54,55,56,57]. * littermate or co-housed mice were used in these studies as controls.

## Supplementary Materials

The following are available online at https://www.mdpi.com/article/10.3390/cancers13081775/s1, Table S1: Anti- and pro-tumorigenic effects of TNF and LT, as implicated by mouse tumor models, Table S2: Nucleotide sequences for used primers, Table S3: Recent clinical trials on cancer treatment involving TNF administration or its blockade.
