# Supplementary material for: Dual Role of TNF and LTα in Carcinogenesis as Implicated by Studies in Mice
Source: Cancers (Basel). 2021 Apr 8;13(8):1775. doi: 10.3390/cancers13081775 (PMC8068266; doi:10.3390/cancers13081775)
Supplement: Supplementary file 1 [file cancers-13-01775-s001.zip › suppl/Table S1.pdf]

Supplementary Materials 1:

Dual role of TNF and LTα in carcinogenesis as implicated by studies in mice

Ekaterina O. Gubernatorova, Almina I. Polinova, Mikhail M. Petropavlovskiy, Olga A. Namakanova, Alexandra D. Medvedovskaya, Ruslan V. Zvartsev, Georgij B. Telegin, Marina S. Drutskaya, Sergei A. Nedospasov

Table S1. Anti- and pro-tumorigenic effects of TNF and LT, as implicated by mouse tumor models

Type of model	Experimental mouse model	Genetic background of recipient mice	Additional experimental procedures	Reported phenotype	PMID
Transplantable mouse tumor model	hTNF-expressing chinese hamster ovarian cells (i.m. or s.c.)	nude mice	None	Reduced tumor growth	[1]
	hTNF-expressing EMT6 mammary carcinoma cells (s.c.)	BALB/c	None	Reduced tumor growth	[2]
	TNF-expressing mammary adenocarcinoma TSA cells				[3]
	mTNF-expressing J558L plasmacytoma cells (s.c.)				[4]
	mTNF-expressing J558L plasmacytoma cells (s.c.)	TNFR1/TNFR2 double knockout in BALB/c	None	No effect (compared with control cells)	[5]
		TNFR1 knockout in BALB/c	None	Reduced tumor growth (compared with control cells)	
	mTNF- or hTNF-expressing J558L plasmacytoma cells (s.c.)	SCID mice	None	Reduced tumor growth	
		TNFR1 knockout in SCID mice	None	Reduced tumor growth for mTNF, no effect for hTNF (compared with control cells)	
	mTNF-expressing EB T lymphoma cells (s.c.)	DBA/2	None	Dose-dependent tumor rejection	[6]
	mTNF-expressing ESB T lymphoma cells (s.c.)		None	Increased hepatic metastases formation	
	mTNF-expressing L929 fibrosarcoma cells (s.c.)	Swiss nude mice	None	Reduced tumor growth and invasiveness	[7]
	hTNF-expressing PRO4L.7 or 8101 UV-induced tumor cells (s.c.)	C57BL/6	None	Reduced tumor growth	[8]
		TNFR1 knockout in C57Bl/6/129	None	No effect (compared with control cells)	
	hTNF-expressing human	nude mice	None	Increased tumor invasiveness	[9]

	ovarian cancer cells (i.p.)				
	hTNF-non-expressing human ovarian cancer cells (i.p.)		None	Reduced tumor growth and invasiveness	
	Meth A sarcoma cells (i.d.)	(BALB/c x C57BL/6)F1 hybrid	Single injection with hTNF (i.p., i.m. or i.t.) 10 days after tumor cell inoculation	Hemorrhagic tumor necrosis	[10]
	hTNF-expressing MCA-205 sarcoma cells (s.c.)	C57BL/6	None	Reduced tumor growth	[11]
	BFS-1 fibrosarcoma cells (i.d.)	tissue-specific LTβ knockout in B or T cells in C57BL/6	None	Reduced tumor growth in both cases	[12]
	mTNF-expressing Py-mT mammary carcinoma cells (under mammary fat pad)	FVB/nJ	None	Increased tumor growth	[13]
	myc-CaP androgen-dependent prostate cancer cells (s.c.)	FVB	Transfer of BM from mice with tissue-specific LTβ knockout in T cells to lethally irradiated wt mice	No effect	[14]
			Transfer of BM from mice with tissue-specific LTβ knockout in B cells to lethally irradiated wt mice	Reduced tumor growth in all cases	
			Multiple injections with sLTβR-Fc fusion protein every 5 days starting 4 days before castration		
	LTβ-specific siRNA-expressing myc-CaP androgen-dependent prostate cancer cells (s.c.)		None		
	EBV-positive nasopharingeal carcinoma xenografts	athymic nude mice	2 injections with hLTβR-specific siRNA (i.t.)	Reduced tumor growth	[15]
	WiDr human colon adenocarcinoma or HT3 human cervical carcinoma cells (s.c.)		Injections with α-hLTβR agonistic Ab (i.p.) every 14 days		[16]
	ID8 murine ovarian carcinoma cells (i.p.)	C57BL/6	Transfer of BM from TNFR1 knockout or TNFR1/TNFR2 double	Reduced tumor burden	[17]

			knockout mice to irradiated wt mice		
		C57BL/6	Transfer of BM from TNFR2 knockout mice to irradiated wt mice	No effect	
		TNFR1 knockout in C57BL/6	None	Reduced tumor burden	
		C57BL/6 with functional TNFR1 only in $CD19^+$ or $LysM^+$ cells	None	Reduced tumor burden	
		C57BL/6 with functional TNFR1 only in $CD4^+$ cells	None	No effect	
	PancTuI, Colo357 or BxPc3 human pancreatic adenocarcinoma cells (orthotypically)	SCID/bg mice	Injections with TNF (i.p.) on days 3, 6, 9, and 12 after tumor cell inoculation	Increased tumor growth and metastasis	[18]
			Multiple injections with etanercept or infliximab (i.p.) after tumor cell inoculation	Reduced tumor growth and metastasis	
			Subtotal pancreatectomy and multiple injections with etanercept or infliximab (i.p.) after tumor cell inoculation	Reduced tumor regrowth and metastasis	
	Human xenografts from bowel or breast carcinomas (s.c.)	athymic nude mice, mixed background	Daily injections with rhTNF (i.p. or i.t.)	Reduced tumor size	[19]
	J558L plasmacytoma, FB61 $TNFR1^{-/-}$ or FD99 $TNFR1/2^{-/-}$ sarcoma cells (s.c.)	TNFR1/TNFR2 double knockout in BALB/c	None	Increased incidence of tumor rejection	[20]
	FB61 $TNFR1^{-/-}$ sarcoma cells (s.c.)	BALB/c	Adoptive transfer of spleen MDSC from TNFR1/TNFR2 double knockout mice to wt mice before tumor cells inoculation	Reduced tumor growth, compared with transfer from wt mice	
		BALB/c	Adoptive transfer of spleen MDSC from wt mice to TNFR1/TNFR2 double knockout mice before tumor cells inoculation	Increased tumor growth, compared with transfer from TNFR1/TNFR2 double knockout mice	
	J558L plasmacytoma, FB61	BALB/c	Injections with α-TNF	Increased incidence of tumor	

	TNFR1$^{-/-}$ sarcoma or TSA mammary adenocarcinoma cells (s.c.)		(i.p.) every 5 days starting at 2 days before tumor inoculation	rejection and reduced tumor growth	
	FB61 TNFR1$^{-/-}$ sarcoma cells (s.c.)	TNFR1 knockout in BALB/c	None	No effect	
		TNFR2 knockout or TNFR1/TNFR2 double knockout in BALB/c	None	Increased incidence of tumor rejection and reduced tumor growth	
Chemically-induced mouse tumor model	MCA-induced sarcoma	TNF knockout in C57BL/6	None	Increased tumor incidence	[21]
		C57BL/6	Transfer of BM from LTα knockout, LIGHT knockout or LTβ/LIGHT double knockout mice to lethally irradiated wt mice	Increased tumor incidence and size	[22]
	MNU/testosterone-induced prostate cancer	TNFR1 knockout in B6.129, C57BL/6 as controls	None	Reduced incidence of adenocarcinoma	[23]
Spontaneous mouse tumor model	Spontaneous lung cancer	CC-LR/TNF-Tg mice, mixed 129SvJ x C57BL/6 background	None	Increased tumor growth and number (compared with CC-LR mice)	[24]
			MDSC depletion	Reduced tumor number (compared with CC-LR/TNF-Tg mice)	
		CC-LR/TNF-KO mice, mixed 129SvJ x C57BL/6 background	None	Reduced tumor growth and number (compared with CC-LR mice)	
	Spontaneous holestatic hepatitis	Mdr-2 deficient mice	Injections with α-TNF (i.p.) for 3 days	Apoptosis of transformed hepatocytes	[25]
	Spontaneous hepatitis and hepatocellular carcinoma (HCC)	TNFR1 knockout in Abl-LT transgenic mice	None	No effect	[26]
		Abl-LT transgenic mice	Multiple injections with α-LTβR antagonistic Ab	Reduced incidence of HCC	
	Spontaneous tumors	TNF knockout, TNFR1 knockout, LTα knockout or TNF/LTα double knockout in p53$^{-/-}$ and p53$^{+/-}$ mice, mixed C57BL/6 background	None	No effect	[27]*
	Spontaneous prostate adenocarcinoma	Heterozygous or homologous deletion of LTα gene in TRAMP mice, C57BL/6 background	None	Reduced tumor incidence, growth and metastasis	[28]*
		TRAMP mice, C57BL/6 background	3 weekly injections with sLTβR-Fc fusion		

			protein (i.p.)		
	Spontaneous intestinal-type gastric tumors	TNF knockout in mice with transgenic expression of *Wnt1*, *Ptgs2* and *Ptges*, mixed background	None	Reduced tumor size, but not number	[29]*
		Mice with transgenic expression of *Wnt1*, *Ptgs2* and *Ptges*, mixed background	Transfer of BM from mice with intact TNF signaling to lethally irradiated TNF knockout mice	Increased tumor size	
		TNFR1 knockout in mice with transgenic expression of *Wnt1*, *Ptgs2* and *Ptges*, mixed background	None	Reduced tumor size	
		Mice with transgenic expression of *Wnt1*, *Ptgs2* and *Ptges*, mixed background	Transfer of BM from TNFR1 knockout mice to lethally irradiated mice with intact TNF signaling		
	Spontaneous T-cell acute lymphoblastic leukaemia	TJ2-Tg in C57BL/6	Multiple injections with neutralizing LTβR-Fc fusion protein (i.p.) for 5 consecutive weeks	Increased survival	[30]
		TJ2-Tg LTβR knockout in C57BL/6	None		

hTNF – human TNF, i.m. – intramuscular, s.c. – subcutaneous, mTNF –mouse TNF, i.p. – intraperitoneal, i.d. – intradermal, i.t. – intratumoral, BM – bone marrow, siRNA – small interfering RNA, *α*- – anti-, hLTβR – human LTβR, rhTNF – recombinant hTNF, MCA – methylcholanthrene, MNU - N-Methyl-N-nitrosourea,

CC-LR/TNF-Tg mice - lung specific K-ras mutant mice with airway epithelial specific TNF overexpression, MDSC - myeloid-derived suppressor cells, CC-LR/TNF-KO mice - lung specific K-ras mutant mice with genetic knockout of TNF, Abl-LT transgenic mice – mice with overexpression of LT*α* and LTβ in liver, TJ2-Tg - ElSRa-TEL-JAK2 transgenic mice. * littermate controls were used in these studies.

References

1. Oliff, A.; Defeo-Jones, D.; Boyer, M.; Martinez, D.; Kiefer, D.; Vuocolo, G.; Wolfe, A.; Socher, S.H. Tumors secreting human TNF/cachectin induce cachexia in mice. *Cell* **1987**, *50*, 555-563, doi:10.1016/0092-8674(87)90028-6.
2. Matory, Y.L.; Dorfman, D.M.; Wu, L.; Chen, M.; Goedegebuure, P.; Eberlein, T.J. Treatment of established tumor is associated with ICAM-1 upregulation and reversed by CD8 depletion in a tumor necrosis factor-alpha gene transfected mouse mammary tumor. *Pathobiology* **1999**, *67*, 186-195, doi:10.1159/000028071.
3. Musiani, P.; Allione, A.; Modica, A.; Lollini, P.L.; Giovarelli, M.; Cavallo, F.; Belardelli, F.; Forni, G.; Modesti, A. Role of neutrophils and lymphocytes in inhibition of a mouse mammary adenocarcinoma engineered to release IL-2, IL-4, IL-7, IL-10, IFN-alpha, IFN-gamma, and TNF-alpha. *Lab Invest* **1996**, *74*, 146-157.
4. Blankenstein, T.; Qin, Z.H.; Uberla, K.; Muller, W.; Rosen, H.; Volk, H.D.; Diamantstein, T. Tumor suppression after tumor cell-targeted tumor necrosis factor alpha gene transfer. *J Exp Med* **1991**, *173*, 1047-1052, doi:10.1084/jem.173.5.1047.

5. Zhao, X.; Mohaupt, M.; Jiang, J.; Liu, S.; Li, B.; Qin, Z. Tumor necrosis factor receptor 2-mediated tumor suppression is nitric oxide dependent and involves angiostasis. *Cancer Res* **2007**, *67*, 4443-4450, doi:10.1158/0008-5472.CAN-07-0185.
6. Qin, Z.; Kruger-Krasagakes, S.; Kunzendorf, U.; Hock, H.; Diamantstein, T.; Blankenstein, T. Expression of tumor necrosis factor by different tumor cell lines results either in tumor suppression or augmented metastasis. *J Exp Med* **1993**, *178*, 355-360, doi:10.1084/jem.178.1.355.
7. Vanhaesebroeck, B.; Mareel, M.; Van Roy, F.; Grooten, J.; Fiers, W. Expression of the tumor necrosis factor gene in tumor cells correlates with reduced tumorigenicity and reduced invasiveness in vivo. *Cancer Res* **1991**, *51*, 2229-2238.
8. Wu, T.H.; Pabin, C.N.; Qin, Z.; Blankenstein, T.; Philip, M.; Dignam, J.; Schreiber, K.; Schreiber, H. Long-term suppression of tumor growth by TNF requires a Stat1- and IFN regulatory factor 1-dependent IFN-gamma pathway but not IL-12 or IL-18. *Journal of immunology* **2004**, *172*, 3243-3251, doi:10.4049/jimmunol.172.5.3243.
9. Kulbe, H.; Thompson, R.; Wilson, J.L.; Robinson, S.; Hagemann, T.; Fatah, R.; Gould, D.; Ayhan, A.; Balkwill, F. The inflammatory cytokine tumor necrosis factor-alpha generates an autocrine tumor-promoting network in epithelial ovarian cancer cells. *Cancer Res* **2007**, *67*, 585-592, doi:10.1158/0008-5472.CAN-06-2941.
10. Pennica, D.; Nedwin, G.E.; Hayflick, J.S.; Seeburg, P.H.; Derynck, R.; Palladino, M.A.; Kohr, W.J.; Aggarwal, B.B.; Goeddel, D.V. Human tumour necrosis factor: precursor structure, expression and homology to lymphotoxin. *Nature* **1984**, *312*, 724-729, doi:10.1038/312724a0.
11. Asher, A.L.; Mule, J.J.; Kasid, A.; Restifo, N.P.; Salo, J.C.; Reichert, C.M.; Jaffe, G.; Fendly, B.; Kriegler, M.; Rosenberg, S.A. Murine tumor cells transduced with the gene for tumor necrosis factor-alpha. Evidence for paracrine immune effects of tumor necrosis factor against tumors. *Journal of immunology* **1991**, *146*, 3227-3234.
12. Daller, B.; Musch, W.; Rohrl, J.; Tumanov, A.V.; Nedospasov, S.A.; Mannel, D.N.; Schneider-Brachert, W.; Hehlgans, T. Lymphotoxin-beta receptor activation by lymphotoxin-alpha(1)beta(2) and LIGHT promotes tumor growth in an NFkappaB-dependent manner. *Int J Cancer* **2011**, *128*, 1363-1370, doi:10.1002/ijc.25456.
13. Li, B.; Vincent, A.; Cates, J.; Brantley-Sieders, D.M.; Polk, D.B.; Young, P.P. Low levels of tumor necrosis factor alpha increase tumor growth by inducing an endothelial phenotype of monocytes recruited to the tumor site. *Cancer Res* **2009**, *69*, 338-348, doi:10.1158/0008-5472.CAN-08-1565.
14. Ammirante, M.; Luo, J.L.; Grivennikov, S.; Nedospasov, S.; Karin, M. B-cell-derived lymphotoxin promotes castration-resistant prostate cancer. *Nature* **2010**, *464*, 302-305, doi:10.1038/nature08782.
15. Or, Y.Y.; Chung, G.T.; To, K.F.; Chow, C.; Choy, K.W.; Tong, C.Y.; Leung, A.W.; Hui, A.B.; Tsao, S.W.; Ng, H.K., et al. Identification of a novel 12p13.3 amplicon in nasopharyngeal carcinoma. *J Pathol* **2010**, *220*, 97-107, doi:10.1002/path.2609.
16. Lukashev, M.; LePage, D.; Wilson, C.; Bailly, V.; Garber, E.; Lukashin, A.; Ngam-ek, A.; Zeng, W.; Allaire, N.; Perrin, S., et al. Targeting the lymphotoxin-beta receptor with agonist antibodies as a potential cancer therapy. *Cancer Res* **2006**, *66*, 9617-9624, doi:10.1158/0008-5472.CAN-06-0217.
17. Charles, K.A.; Kulbe, H.; Soper, R.; Escorcio-Correia, M.; Lawrence, T.; Schultheis, A.; Chakravarty, P.; Thompson, R.G.; Kollias, G.; Smyth, J.F., et al. The tumor-promoting actions of TNF-alpha involve TNFR1 and IL-17 in ovarian cancer in mice and humans. *J Clin Invest* **2009**, *119*, 3011-3023, doi:10.1172/JCI39065.
18. Egberts, J.H.; Cloosters, V.; Noack, A.; Schniewind, B.; Thon, L.; Klose, S.; Kettler, B.; von Forstner, C.; Kneitz, C.; Tepel, J., et al. Anti-tumor necrosis factor therapy inhibits pancreatic tumor growth and metastasis. *Cancer Res* **2008**, *68*, 1443-1450, doi:10.1158/0008-5472.CAN-07-5704.
19. Balkwill, F.R.; Lee, A.; Aldam, G.; Moodie, E.; Thomas, J.A.; Tavernier, J.; Fiers, W. Human tumor xenografts treated with recombinant human tumor necrosis factor alone or in combination with interferons. *Cancer Res* **1986**, *46*, 3990-3993.
20. Zhao, X.; Rong, L.; Zhao, X.; Li, X.; Liu, X.; Deng, J.; Wu, H.; Xu, X.; Erben, U.; Wu, P., et al. TNF signaling drives myeloid-derived suppressor cell accumulation. *J Clin Invest* **2012**, *122*, 4094-4104, doi:10.1172/JCI64115.
21. Swann, J.B.; Vesely, M.D.; Silva, A.; Sharkey, J.; Akira, S.; Schreiber, R.D.; Smyth, M.J. Demonstration of inflammation-induced cancer and cancer immunoediting during primary tumorigenesis. *Proceedings of the National Academy of Sciences of the United States of America* **2008**, *105*, 652-656, doi:10.1073/pnas.0708594105.
22. Hu, X.; Zimmerman, M.A.; Bardhan, K.; Yang, D.; Waller, J.L.; Liles, G.B.; Lee, J.R.; Pollock, R.; Lev, D.; Ware, C.F., et al. Lymphotoxin beta receptor mediates caspase-dependent tumor cell apoptosis in vitro and tumor suppression in vivo despite induction of NF-kappaB activation. *Carcinogenesis* **2013**, *34*, 1105-1114, doi:10.1093/carcin/bgt014.

23. Galheigo, M.R.; Cruz, A.R.; Cabral, A.S.; Faria, P.R.; Cordeiro, R.S.; Silva, M.J.; Tomiosso, T.C.; Goncalves, B.F.; Pinto-Fochi, M.E.; Taboga, S.R., et al. Role of the TNF-alpha receptor type 1 on prostate carcinogenesis in knockout mice. *Prostate* **2016**, *76*, 917-926, doi:10.1002/pros.23181.
24. Gong, L.; da Silva Caetano, M.; Cumpian, A.M.; Daliri, S.; Garza Flores, A.; Chang, S.H.; Ochoa, C.E.; Evans, C.M.; Yu, Z.; Moghaddam, S.J. Tumor necrosis factor links chronic obstructive pulmonary disease and K-ras mutant lung cancer through induction of an immunosuppressive pro-tumor microenvironment. *Oncoimmunology* **2016**, *5*, e1229724, doi:10.1080/2162402X.2016.1229724.
25. Pikarsky, E.; Porat, R.M.; Stein, I.; Abramovitch, R.; Amit, S.; Kasem, S.; Gutkovich-Pyest, E.; Urieli-Shoval, S.; Galun, E.; Ben-Neriah, Y. NF-kappaB functions as a tumour promoter in inflammation-associated cancer. *Nature* **2004**, *431*, 461-466, doi:10.1038/nature02924.
26. Haybaeck, J.; Zeller, N.; Wolf, M.J.; Weber, A.; Wagner, U.; Kurrer, M.O.; Bremer, J.; Iezzi, G.; Graf, R.; Clavien, P.A., et al. A lymphotoxin-driven pathway to hepatocellular carcinoma. *Cancer Cell* **2009**, *16*, 295-308, doi:10.1016/j.ccr.2009.08.021.
27. Kuprash, D.V.; Qin, Z.; Ito, D.; Grivennikov, S.I.; Abe, K.; Drutskaya, L.N.; Blankenstein, T.; Nedospasov, S.A. Ablation of TNF or lymphotoxin signaling and the frequency of spontaneous tumors in p53-deficient mice. *Cancer letters* **2008**, *268*, 70-75, doi:10.1016/j.canlet.2008.03.023.
28. Zhou, P.; Fang, X.; McNally, B.A.; Yu, P.; Zhu, M.; Fu, Y.X.; Wang, L.; Liu, Y.; Zheng, P. Targeting lymphotoxin-mediated negative selection to prevent prostate cancer in mice with genetic predisposition. *Proceedings of the National Academy of Sciences of the United States of America* **2009**, *106*, 17134-17139, doi:10.1073/pnas.0905707106.
29. Oshima, H.; Ishikawa, T.; Yoshida, G.J.; Naoi, K.; Maeda, Y.; Naka, K.; Ju, X.; Yamada, Y.; Minamoto, T.; Mukaida, N., et al. TNF-alpha/TNFR1 signaling promotes gastric tumorigenesis through induction of Noxo1 and Gna14 in tumor cells. *Oncogene* **2014**, *33*, 3820-3829, doi:10.1038/onc.2013.356.
30. Fernandes, M.T.; Ghezzo, M.N.; Silveira, A.B.; Kalathur, R.K.; Povoa, V.; Ribeiro, A.R.; Brandalise, S.R.; Dejardin, E.; Alves, N.L.; Ghysdael, J., et al. Lymphotoxin-beta receptor in microenvironmental cells promotes the development of T-cell acute lymphoblastic leukaemia with cortical/mature immunophenotype. *Br J Haematol* **2015**, *171*, 736-751, doi:10.1111/bjh.13760.
