# Supplementary material for: Dual Role of TNF and LTα in Carcinogenesis as Implicated by Studies in Mice
Source: Cancers (Basel). 2021 Apr 8;13(8):1775. doi: 10.3390/cancers13081775 (PMC8068266; doi:10.3390/cancers13081775)
Supplement: Supplementary file 1 [file cancers-13-01775-s001.zip › suppl/Table S2.pdf]

Supplementary Materials 2:

Dual role of TNF and LTα in carcinogenesis as implicated by studies in mice

Ekaterina O. Gubernatorova, Almina I. Polinova, Mikhail M. Petropavlovskiy, Olga A. Namakanova, Alexandra D. Medvedovskaya, Ruslan V. Zvartsev, Georgij B. Telegin, Marina S. Drutskaya, Sergei A. Nedospasov

Acute skin inflammation was initiated in LTα-deficient and control mouse skin by administration of 4 mcg PMA dissolved in 100 mcl acetone on the shaved back. Then, starting from day 2, same amounts of PMA were administered onto every animal, 10 minutes later this area was covered with 25 mcg Aldara cream, with such operation to be performed each two days until termination day, varying between 4 and 6 administering acts overall. Two hours after the last PMA or Aldara application all mice were euthanized. A small part of either healthy or damaged skin near 10 mm diameter, was taken and put into RNALater Stabilization Solution to be later transported into 700 mcl TRK Lysis Buffer to perform homogenization using blade homogenizer, after what total RNA was extracted from samples with E.Z.N.A.® Total RNA Kit using OMEGA BIO-TEK standard protocol. All RNA samples were cleaned from genome DNA by incubation with DNAse at 37°C for 30 minutes, afterwards the reaction was stopped by incubation with 50 mM EDTA at 65°C for 10 minutes. First cDNA chain synthesis was performed using RevertAid First Strand cDNA Synthesis kit reagents with hexamer primers. Quantitative real-time PCR was performed using Evrogen SYBR Green reagents, including EVA Green intercalating dye, reaction scheme shown below: 95 °C 15 sec (duplet denaturation) → 62 °C 30 sec (primer annealing) → 72 °C 20 sec (elongation). Primers were selected using PrimerBlast having exon joint annealing as a criteria and synthetized by Evrogen.

Table S2. Nucleotide sequences for used primers.

Gene product name	Sequence	
	Forward	Reverse
B-actin	GATCTCTATGCCAACACAGT	AGAAAGGGTGTAAAACGCAG
Tnf	TCTGTCTACTGAACTTCGGG	TTGGTGGTTTGCTACGAC
Lta	ATGGCATCCTGAAACCTGCTGC	GGGAGTTGTTGCTCAAAGAGAAGC
Ltb	AATCTAGCCTCCACATCCCA	CATCCAAGCGCCTATGAG
Cxcl1	AACCGAAGTCATAGCCACAC	GAGCAGTCTGTCTTCTTTCTCC
Il23	CGGGACATATGAATCTACTAAGAG	GTTGTCCTTGAGTCCTTGTG
Il13	GCTGAGCAACATCACACAAG	ACAGAGGCCATGCAATATCC
Il6	CTCTGCAAGAGACTTCCATCC	TTCTGCAAGTGCATCATCGT
Il10	GACAATAACTGCACCCACTTCC	AACCCAAGTAACCCTTAAAGTCC
S100a9	TAGCCTTGAGCAAGAAGATGG	CTTCCACCATTTGTCTGAATTCC
S100a8	CCTTTGTCAGCTCCGTCTTCA	TCCAGTTCAGACGGCATTGT
Defensin b	TTCAAGCCTCATCTGTCAGCC	GTGAGAATGCCAACACCTGGC
Cathelecidin	CTTCAAGGAACAGGGGGTGG	ACCTTTGCGGAGAAGTCCAG
