# Supplementary material for: Dual Role of TNF and LTα in Carcinogenesis as Implicated by Studies in Mice
Source: Cancers (Basel). 2021 Apr 8;13(8):1775. doi: 10.3390/cancers13081775 (PMC8068266; doi:10.3390/cancers13081775)
Supplement: Supplementary file 1 [file cancers-13-01775-s001.zip › suppl/Table S3.pdf]

Supplementary Materials 3:

Dual role of TNF and LTα in carcinogenesis as implicated by studies in mice

Ekaterina O. Gubernatorova, Almina I. Polinova, Mikhail M. Petropavlovskiy, Olga A. Namakanova, Alexandra D. Medvedovskaya, Ruslan V. Zvartsev, Georgij B. Telegin, Marina S. Drutskaya, Sergei A. Nedospasov

Table S3. Recent clinical trials on cancer treatment involving TNF administration or its blockade

Type of cancer	Target cytokine/ cytokine receptor	Drug/ treatment	Additional drugs/ treatments	Outcome	Ref.	NCT trial number and year of study start
Melanoma	TNF	rhTNF	Melphalan, ILP	• Phase III trial: The addition of TNF to melphalan did not demonstrate a significant enhancement of short-term response rates over melphalan alone by the 3-month follow-up.<br>• TNF plus melphalan was associated with a higher complication rate.	[1]	NCT00003789 (1999)
			Melphalan, IFNγ , hyperthermic ILP	• Phase III trial: ILP with TNF/IFNγ/melphalan is more effective (CR rate was 72%) then ILP with only melphalan (CR rate was 58%).<br>• ILP with TNF/IFNγ/melphalan can be safely performed with mostly mild side effects.	[2,3]	NCT00001296 (1992)
		L19TNF (recombinant fusion protein that consists of fully human recombinant monoclonal antibody (L19) and the human TNF)	L19IL2	• Phase II trial: Intralesional injection of L19-IL2 and L19-TNF slowed down metastases progression.<br>• Treatment was well tolerated, with mostly mild side effects limited to injection site.	[4]	NCT02076633 (2012)
Colorectal Cancer	TNF		No	• Phase I/II trial: No objective tumor responses.<br>• The maximally tolerated dose (MTD) was not reached. Intravenous L19-TNF was safe up to 13 μg/kg.	[5]	NCT01253837 (2007)

Colorectal Cancer, Head and Neck Cancer, Kidney Cancer	TNF	NGR-hTNF (CNGRC peptide-TNF conjugate)	No	• Phase I trial: No objective responses were observed.<br>• NGR-hTNF was well tolerated. The MTD was 45 μg/m^2.	[6]	NCT00098943 (2004)
Diffuse large B-cell lymphoma	TNF		Rituximab, doxorubicin, cyclophosphamide, vincristine, prednisone	• Phase II trial: Fast and prominent tumor regression was observed in 9 of the 12 assessed patients. NGR-hTNF selectively increased vascular permeability in tumoral/peritumoral areas.<br>• The NGR-hTNF/R-CHOP* combination was well tolerated.	[7]	NCT03536039 (2016)
Hematological cancers	Anti-TNF, anti-LTα_3	Etanercept (fusion protein that consists of extracellular binding domain of the 75kD TNFR2 and the constant portion of human IgG1)	Methylpredni-solone	• Phase II trial: Etanercept in combination with corticosteroids (methylprednisolone) was associated with high response rates and overall survival in children with IPS.<br>• Etanercept was well tolerated.	[8]	NCT00309907 (2006)
Myelodysplastic Syndromes	Anti-TNF, anti-LTα_3		Azacitidine	• Phase I/II trial: Etanercept increased the response rate and the duration of response observed with azacitidine therapy.<br>• Most of the side effects were qualified as mild.	[9]	NCT00118287 (2005)
Non-melanoma Skin Cancer	TNF	L19TNF (recombinant fusion protein that consists of fully human recombinant monoclonal antibody (L19) and the human TNF)	L19IL2	–	NP	NCT04362722 (2020)
Melanoma	Anti-TNF	Certolizumab (PEGylated humanized antigen-binding fragment (Fab’) of an anti-TNF monoclonal antibody) [10]<br>Infliximab (chimeric monoclonal antibody)	Nivolumab, Ipilimumab	–	NP	NCT03293784 (2017)

TNF – tumor necrosis factor, rhTNF – recombinant human tumor necrosis factor, ILP – isolated limb perfusion, IFNγ – interferon-gamma, CR – complete responses, hTNF – human tumor necrosis factor, MTD – maximum tolerated dose, LTα_3 – lymphotoxin α, IPS - idiopathic pneumonia syndrome, NP – not published.

*R-CHOP is an acronym that stands for combination of drugs used in chemotherapy: R – Rituximab, C – Cyclophosphamide, H – Doxorubicin Hydrochloride (Hydroxydaunomycin), O – Vincristine Sulfate (Oncovin), P – Prednison.

References

1. Cornett, W.R.; McCall, L.M.; Petersen, R.P.; Ross, M.I.; Briele, H.A.; Noyes, R.D.; Sussman, J.J.; Kraybill, W.G.; Kane, J.M., 3rd; Alexander, H.R., et al. Randomized multicenter trial of hyperthermic isolated limb perfusion with melphalan alone compared with melphalan plus tumor necrosis factor: American College of Surgeons Oncology Group Trial Z0020. *J Clin Oncol* **2006**, *24*, 4196-4201, doi:10.1200/JCO.2005.05.5152.
2. Thom, A.K.; Alexander, H.R.; Andrich, M.P.; Barker, W.C.; Rosenberg, S.A.; Fraker, D.L. Cytokine levels and systemic toxicity in patients undergoing isolated limb perfusion with high-dose tumor necrosis factor, interferon gamma, and melphalan. *J Clin Oncol* **1995**, *13*, 264-273, doi:10.1200/JCO.1995.13.1.264.
3. Fraker, D.L.; Alexander, H.R.; Ross, M.; Bartlett, D.L.; Tyler, D.; Libutti, L.S.; Boddie, A.; Briele, H.; Karakousis, G. A phase III trial of isolated limb perfusion for extremity melanoma comparing melphalan alone versus melphalan plus tumor necrosis factor (TNF-α) plus interferon-gamma (IFN). *Annals of Surgical Oncology* **2002**, *9*, S8, doi:10.1007/BF02640542.
4. Danielli, R.; Patuzzo, R.; Di Giacomo, A.M.; Gallino, G.; Maurichi, A.; Di Florio, A.; Cutaia, O.; Lazzeri, A.; Fazio, C.; Miracco, C., et al. Intralesional administration of L19-IL2/L19-TNF in stage III or stage IVM1a melanoma patients: results of a phase II study. *Cancer Immunol Immunother* **2015**, *64*, 999-1009, doi:10.1007/s00262-015-1704-6.
5. Spitaleri, G.; Berardi, R.; Pierantoni, C.; De Pas, T.; Noberasco, C.; Libbra, C.; Gonzalez-Iglesias, R.; Giovannoni, L.; Tasciotti, A.; Neri, D., et al. Phase I/II study of the tumour-targeting human monoclonal antibody-cytokine fusion protein L19-TNF in patients with advanced solid tumours. *J Cancer Res Clin Oncol* **2013**, *139*, 447-455, doi:10.1007/s00432-012-1327-7.
6. van Laarhoven, H.W.; Fiedler, W.; Desar, I.M.; van Asten, J.J.; Marreaud, S.; Lacombe, D.; Govaerts, A.S.; Bogaerts, J.; Lasch, P.; Timmer-Bonte, J.N., et al. Phase I clinical and magnetic resonance imaging study of the vascular agent NGR-hTNF in patients with advanced cancers (European Organization for Research and Treatment of Cancer Study 16041). *Clin Cancer Res* **2010**, *16*, 1315-1323, doi:10.1158/1078-0432.CCR-09-1621.
7. Ferreri, A.J.M.; Calimeri, T.; Conte, G.M.; Cattaneo, D.; Fallanca, F.; Ponzoni, M.; Scarano, E.; Curnis, F.; Nonis, A.; Lopedote, P., et al. R-CHOP preceded by blood-brain barrier permeabilization with engineered tumor necrosis factor-alpha in primary CNS lymphoma. *Blood* **2019**, *134*, 252-262, doi:10.1182/blood.2019000633.
8. Yanik, G.A.; Grupp, S.A.; Pulsipher, M.A.; Levine, J.E.; Schultz, K.R.; Wall, D.A.; Langholz, B.; Dvorak, C.C.; Alangaden, K.; Goyal, R.K., et al. TNF-receptor inhibitor therapy for the treatment of children with idiopathic pneumonia syndrome. A joint Pediatric Blood and Marrow Transplant Consortium and Children's Oncology Group Study (ASCT0521). *Biol Blood Marrow Transplant* **2015**, *21*, 67-73, doi:10.1016/j.bbmt.2014.09.019.
9. Scott, B.L.; Ramakrishnan, A.; Storer, B.; Becker, P.S.; Petersdorf, S.; Estey, E.H.; Deeg, H.J. Prolonged responses in patients with MDS and CMML treated with azacitidine and etanercept. *Br J Haematol* **2010**, *148*, 944-947, doi:10.1111/j.1365-2141.2009.08061.x.
10. Goel, N.; Stephens, S. Certolizumab pegol. *MAbs* **2010**, *2*, 137-147, doi:10.4161/mabs.2.2.11271.
